# Differential Roles of CD36 in Regulating Muscle Insulin Response Depend on Palmitic Acid Load

**DOI:** 10.3390/biomedicines11030729

**Published:** 2023-02-28

**Authors:** Jingyu Sun, Yajuan Su, Jiajia Chen, Duran Qin, Yaning Xu, Hang Chu, Tianfeng Lu, Jingmei Dong, Lili Qin, Weida Li

**Affiliations:** Sports and Health Research Center, Department of Physical Education, Translational Medical Center for Stem Cell Therapy and Institute for Regenerative Medicine, Shanghai East Hospital, School of Life Sciences and Technology, Tongji University, Shanghai 200092, China

**Keywords:** CD36, insulin signaling, mitochondrial dysfunction, ER stress, C2C12 myotubes

## Abstract

The possible role of fatty acid translocase (CD36) in the treatment of obesity has gained increasing research interest since researchers recognized its coordinated function in fatty acid uptake and oxidation. However, the effect of CD36 deficiency on intracellular insulin signaling is complex and its impact may depend on different nutritional stresses. Therefore, we investigated the various effects of CD36 deletion on insulin signaling in C2C12 myotubes with or without palmitic acid (PA) overload. In the present work, we reported the upregulated expression levels of CD36 in the skeletal muscle tissues of obese humans and mice as well as in C2C12 myotubes with PA stimulation. CD36 knockdown using RNA interference showed that insulin signaling was impaired in CD36-deficient C2C12 cells in the absence of PA loading, suggesting that CD36 is essential for the maintenance of insulin action, possibly resulting from increased mitochondrial dysfunction and endoplasmic reticulum (ER) stress; however, CD36 deletion improved insulin signaling in the presence of PA overload due to a reduction in lipid overaccumulation. In conclusion, we identified differential roles of CD36 in regulating muscle insulin response under conditions with and without PA overload, which provides supportive evidence for further research into therapeutic approaches to diabetes.

## 1. Introduction

Obesity is one of the most serious public health diseases in the world because it predisposes multiple diseases and shortens life expectancy. Obesity is considered an important link in type 2 diabetes and is likely caused by insulin resistance (IR) [1]. Skeletal muscle is responsible for glucose disposal and is essential to systemic glucose regulation [2]. Therefore, muscle (IR is a major feature of obesity and type 2 diabetes [3]. Phosphorylation of AKT is thought to be a critical step in intracellular insulin signaling [4,5]. Defective insulin-induced AKT phosphorylation is associated with obesity-related muscle insulin resistance [6,7].

CD36, as a transmembrane glycoprotein, is abundantly expressed in adipocytes, macrophages, muscle cells, and hepatocytes [8]. CD36 promotes the uptake and oxidation of long-chain fatty acids (LCFAs), which in turn regulate intracellular metabolic homeostasis [9]. Recently, the potential regulatory role of CD36 on insulin signaling has been increasingly investigated; however, the effect of CD36 on insulin signaling is complex [10,11]. Some studies have shown that upregulation of CD36 is associated with insulin resistance, suggesting that CD36 may be a negative mediator of insulin sensitivity [11]. However, other studies show that CD36 has an opposite regulatory effect on insulin sensitivity [12]. Collectively, the exact regulation of insulin sensitivity by CD36 is highly controversial and the underlying mechanisms remain uncovered. Interestingly, it has been shown that CD36-deficient mice clear glucose more rapidly on a chow diet; however, after switching to a high fructose diet, CD36-deficient mice exhibited significant glucose intolerance compared to wild-type (WT) mice [13], hypothesizing that the paradoxical role of CD36 in insulin signaling may depend on different nutritional stresses. Here, we investigated the effect of CD36 on insulin signaling in C2C12 myotubes under different nutritional conditions with or without PA overload.

Mitochondrial dysfunction is commonly observed in the skeletal muscle of mice with IR [14]. The presence of reduced mitochondrial protein expression, decreased oxygen consumption rates, impaired ATP synthesis, and reduced mitochondrial protein expression have been observed in people with obesity or IR [15,16]. Recent research also proposes that ER stress has emerged as a key player in the onset of insulin resistance [17]. Considering that CD36 regulates insulin action, mitochondrial dysfunction and ER stress may be key regulators of insulin signaling by CD36 [18].

In the present study, the role of CD36 in the skeletal muscle tissue of obese humans and mice and in C2C12 myotubes under PA stimulation was first examined. To further determine the effect of CD36 deficiency on insulin signaling under different nutritional conditions, the cultured C2C12 myotube models with or without PA overload were utilized. Finally, mitochondrial dysfunction and ER stress were highlighted as potential mechanisms by which CD36 may regulate insulin signaling.

## 2. Materials and Methods

### 2.1. Animal

Eight-week-old male C57BL/6 mice were provided by Charles River Laboratories (Beijing, China). All mice in the study were randomly divided into the following two groups: a normal diet group (ND) (10% fat, SLACOM, Shanghai, China) and a high fat diet group (HFD) (40% fat, SLACOM, Shanghai, China). Mice were housed in comfortable conditions with a 12:12 h light–dark cycle, at a temperature of 20–26 °C, and with free access to feeding and drinking. After 4 months of high-fat feeding, mice were euthanized and gastrocnemius muscle tissues were collected, rapidly frozen in liquid nitrogen, and stored at −80 °C for subsequent analysis. All animal experiments were approved by the Animal Care and Use Committee of Tongji University.

### 2.2. Cell Culture

Mouse skeletal muscle cell line (C2C12) was cultured with growth medium (high-glucose DMEM supplemented with 10% FBS (#10270106, Thermo Fisher Scientific, Waltham, MA, USA) and L-glutamine (2 mmol/L, #25030081, Thermo Fisher Scientific, Waltham, Massachusetts, USA). C2C12 cells were differentiated in differentiation medium (low-glucose DMEM supplemented with 2% horse serum (#C2510, VivaCell Biosciences, Shanghai, China)). Differentiated C2C12 myotubes were starved (16–18 h) in buffer A (low-glucose DMEM supplemented with 2 mmol/L L-glutamine, 100 mmol/L MEM nonessential amino acids (#11140050, Thermo Fisher Scientific, Waltham, MA, USA), 100 units/mL penicillin, and 100 mg/mL streptomycin (#25030081, YuanPei, Shanghai, China)). For PA treatment, the fatty acid (FA)-bovine serum albumin (BSA) conjugate was prepared as follows: firstly, PA was dissolved in sodium hydroxide solution at 95 °C to a final concentration of 100 mmol/L. Secondly, the 100 mmol/L PA solution was diluted to 10 mmol/L with BSA solution. The stock solution of PA or BSA were mixed into culture media at a final concentration of 200 μM. FA-free BSA was obtained from Equitech-Bio (#BAH66, Kerrville, TX, USA). Palmitate acid was obtained from Sigma–Aldrich (#O1383, St. Louis, MO, USA).

### 2.3. RNA Interference

siRNA duplexes that target mouse CD36 (siCD36-1: 5′-GGAUGACAACUUCACAGUUTT-3′; siCD36-2: 5′-GGAUUGGAGUGGUGAUGUUTT-3′) and negative control (siCont, 5′-UUCUCCGAACGUGUCACGUTT-3′) were synthesized by Sigma–Aldrich. Lipofectamine RNAiMAX (#13778075, Thermo Fisher Scientific, Waltham, MA, USA) was used to transfect C2C12 cells with siRNA at a final concentration of 20 nM according to the manufacturer’s protocol. All siRNA-transfected myotubes had normal morphology and were used 72 h after transfection. Western blot analysis confirmed the transfection efficiency.

### 2.4. CD36 Plasmids Construction

Wild-type mouse skeletal muscle RNA was extracted and reversed to cDNA; primers were designed to amplify the CD36 CDS sequence from cDNA. Then CD36 overexpression sequence was cloned into the PWPI vector to obtain CD36 overexpression plasmids. CD36 plasmid was packaged to lentivirus and transfected with C2C12 for the next analysis.

### 2.5. Transcriptome Sequencing

Total RNA from the two groups was extracted with TRIzol reagent (Invitrogen, Carlsbad, CA, USA). A cDNA library was constructed using the Illumina NovaseqTM 6000 sequence platform (LC Bio, Hangzhou, China). By using the Illumina paired-end RNA- sequencing approach, the transcriptome was sequenced, generating one million 2 x 150 bp paired-end reads from the sample, which were dependent on Illumina paired-end RNA- sequencing. Reads from all samples were compared to the reference genome by using the HISAT (version 2.0, Johns Hopkins University, Baltimore, MD, USA) software package. After calculation with StringTie software (Johns Hopkins University, Baltimore, MD, USA), FPKM values were generated to indicate the expression levels of the mRNAs. Genes were subjected to differential expression analysis by using edgeR software between two different groups. Differentially expressed genes (DEGs) were considered at *p* value < 0.05 and absolute log2 (fc) ≥ 1. Significantly, DEGs were enriched to KEGG pathways by using the OmicStudio tools (LC Bio, Hangzhou, China). The raw RNA-seq data has been uploaded to the public database of GSE under accession number: GSE204686. The KEGG pathways were identified as significantly enriched following a hypergeometric test (FDR < 0.05). For the reanalysis of public human transcription data, raw data were downloaded from the GSE database (no. GSE81965). After quality analysis with fastp (version 0.20.1, HaploX, Shenzhen, China), sample reads were mapped to the reference genome of humans (version GRCh38) using STAR (version 2.7.6a, NHGRI, National Institutes of Health, Bethesda, MD, USA). The reads count values after calculation with htseq (version 0.13.5) represent the expression level of CD36 between obese individuals and non-obese controls.

### 2.6. Oil Red O Staining

C2C12 myotubes were cultivated on coverslips in six-well plates with 200 μM PA for 16 h to stain intracellular lipid deposits. The cells were then stained with Oil Red O (#O0625, Sigma Aldrich, Shanghai, China) after being fixed with 4% paraformaldehyde. A microscope was used to capture images.

### 2.7. Triglyceride (TG) Content Assay

Intracellular TG content in C2C12 myotube cells was determined with a Tissue TG Content Assay Kit (#E1013, Applygen, Beijing, China). 1 × 10^6^ myotube cells were washed twice with DPBS and lysed with lysis buffer. The levels of TG released from myotube cells were analyzed according to the instructions of the kit and normalized by total protein quantity.

### 2.8. Reactive Oxygen Species (ROS) Measurement

ROS measurement was performed according to the protocol of the ROS assay kit (#E004-1-1, Nanjing Jiancheng Bioengineering Institute, Jiangsu, China). After treatment, the cells were placed in 96-well black opaque plates and incubated with 25 M DCFH-DA for 30 min in the dark in a 37 °C incubator. The fluorescence spectrum was recorded at an excitation wavelength of 488 nm and an emission wavelength of 525 nm (SpectraMax M5, Molecular Devices, Silicon Valley, CA, USA).

### 2.9. Adenosine Triphosphatase (ATPase) Activity Assay

The activity of ATPase was determined using corresponding microplate assay kits (#A070-1-1, Nanjing Jiancheng Bioengineering Institute, Jiangsu, China) according to the kits’ protocol. A TECAN microplate reader (TECAN, Sunrise, Mannedorf, Switzerland) was used to record the absorbance.

### 2.10. Transmission Electron Microscopy (TEM)

C2C12 cells were collected after centrifugation. The TEM fixative buffer containing 2% glutaraldehyde, 0.1 M sodium cacodylate, 0.5% polyformaldehyde, 3 mM CaCl_2_, and 0.1 M sucrose was used to fix the cell sample at 4 °C for 2–4 h. Then, the differentiated C2C12 cells were washed thrice with PBS for 15 min each, dehydrated in series concentration of ethanol and in acetone, and inserted into a mixture buffer of equal volume of acetone and EMBed 812 overnight at 37 °C. Pure EMBed 812 was held at 37 °C overnight. The slides were stained twice with 2% uranium acetate saturated alcohol solution and 2.6% lead citrate and examined at 80 kV by using Tecnai 10 TEM (TECNAI G2 F20 S-TWIN, FEI, Hillsboro, OR, USA). The ultrastructure of the mitochondria was observed using a Hitachi (HT7800) transmission electron microscope.

### 2.11. Quantitative RT-PCR

TRIzol (Invitrogen, Carlsbad, CA, USA) reagent was used to isolate the total RNA. The RNA was fully released after 5 min at room temperature and then added 100 μL of chloroform for RNA extraction. About 160 μL of the upper aqueous phase was aspirated after centrifugation at 12,000 rpm for 15 min at 4 °C. A total of 160 μL of isopropanol was added, and the solution was well-mixed for 10 min at room temperature. After the RNA pellet was washed and dissolved, nanodrop was used to determine the concentration and quality of the RNA sample. The quality RNA was reversed to cDNA according to the instructions (#KR106-02, TIANGEN, Beijing, China) and subjected to PCR reaction. The procedure of PCR was used to determine gene expression levels as previously reported [19]. The calculation formula of 2^−△△Ct^ was used to assess the expression levels of target genes. We used GAPDH as the internal reference in this study. The sequences of primers are summarized in Table 1.

### 2.12. Western Blot Analysis

RIPA buffer (1% Triton, 20 mM Tris-HCl at pH 7.5, 150 mM NaCl, 1 mM Na_3_VO_4_, and 50 mM NaF) with proteases and a phosphatase inhibitors cocktail (Roche Diagnostics, Germany) was used to lyse the sample. A BCA test (Thermo Scientific, Waltham, MA, USA) was used to measure the concentration of protein. Protein samples were loaded in SDS-PAGE, and the gels were transferred to the polyvinylidene difluoride membranes (Millipore Corp., Bedford, MA, USA). The membranes were immersed in a blocking reagent (5% non-fat milk) for 1 h at room temperature. After washing with TBST, the membranes were incubated with primary antibodies, including rabbit antibody phospho-AKT (P-AKT, S473) (#4060), total AKT (T-AKT) (#ab187783), phospho-p44/42 MAPK (P-ERK, T202/Y204) (#4370), total ERK (T-ERK) (#4695), cytochrome c (Cyto-C, #4280), cytochrome c oxidase subunit 4 (COX-4, #4850T), glucose-regulated protein 78 (GRP-78, #3177), C/EBP homologous protein (CHOP, #2895T) purchased from Cell Signaling Technology (Danvers, MA, USA), goat antibody anti CD36 (CD36, #AF2519) purchased from R&D system (Minneapolis, MN, USA), and glyceraldehyde-3-phosphate dehydrogenase (GAPDH, #AB0037) from Abways (Shanghai, China). The membranes were washed thrice after 1 h of incubation with peroxidase-conjugated secondary antibodies from Yeasen (Shanghai, China). Target protein bands were detected by ECL luminescence technique and analyzed using Image J software (V1.8.0, National Institutes of Health, Bethesda, MD, USA) for grayscale values.

### 2.13. Statistical Analysis

Data were presented as mean ± SEM. For multiple comparisons, statistical significance was established using paired Student t test or one-way ANOVA. The level of significance was set to *p* < 0.05. SPSS 19.0 (Chicago, IL, USA) was used for data analysis.

## 3. Results

### 3.1. CD36 Is Highly Expressed in Skeletal Muscle under the Condition of HFD and PA Treatment

**Figure 1:**
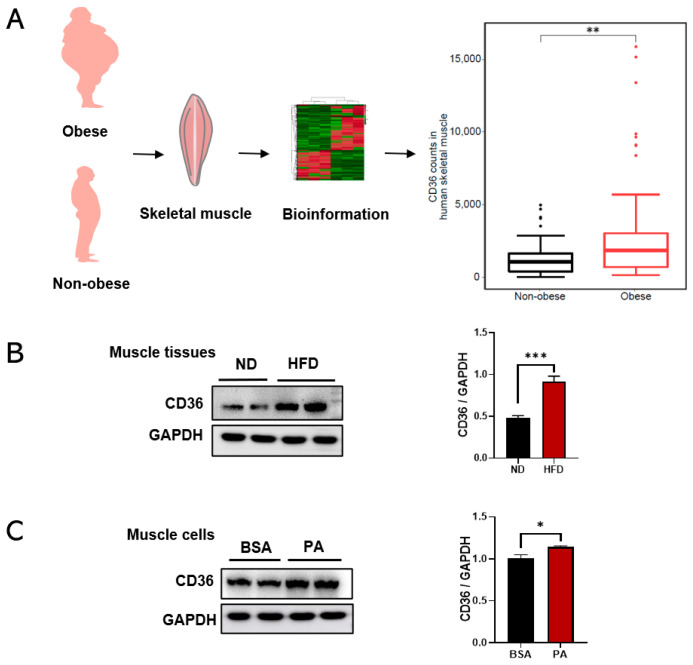
The expression levels of CD36 are upregulated in the skeletal muscle tissues of obese humans and mice in vivo and in vitro. (**A**) Bioinformatics analysis of CD36 counts in human skeletal muscles in the obese and non-obese groups. (**B**) Western blot analysis and quantification of CD36 protein expression in the skeletal muscles of HFD-fed mice. (**C**) Western blot analysis and quantification of CD36 protein expression in skeletal muscles cells under PA stimulation. CD36 expression was normalized to that of the GAPDH gene. Values are presented as the mean ± SEM of three independent experiments. * *p* < 0.05, ** *p* < 0.01, *** *p* < 0.001 versus the siCont group.

CD36 is correlated with the progression of metabolic diseases [11]. To investigate the relevance of obesity and CD36 expression, this study processed and reanalyzed publicly available transcriptome sequencing data (GEO ID: GSE81965) from the human skeletal muscle of obese and non-obese controls. Bioinformatics analysis showed that CD36 expression was upregulated in skeletal muscles from obese individuals compared with non-obese controls (Figure 1A), which were also validated in an HFD-induced animal model. The results showed that the expression level of CD36 was increased in the skeletal muscle tissue of HFD-induced mice (Figure 1B). To further test the effect of obesity and its complications on CD36 expression in the skeletal muscle cells, we established a suitable culture condition by differentiating C2C12 cells with a PA-containing medium, which mimics HFD conditions in vitro. The result also showed an increase in CD36 protein expression in PA-stimulated C2C12 myotubes (Figure 1C). Hence, it is hypothesized that CD36 may be associated with metabolic disorders in the skeletal muscle of obese humans and mice as well as in PA-stimulated C2C12 myotubes.

### 3.2. Loss of CD36 Impairs Insulin Signaling in C2C12 Myotubes in the Absence of PA Loading

**Figure 2:**
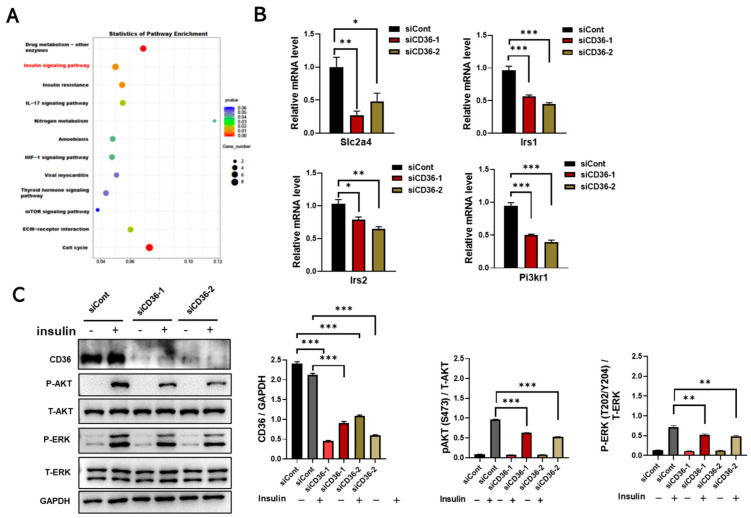
Loss of CD36 impairs insulin signaling in C2C12 myotubes without PA loading. (**A**) KEGG pathway analysis of differentiated C2C12 cells after transfection with scrambled siCont or siCD36. (**B**) Gene expression of Slc2a4, Irs1, Irs2, and Pi3kr1 in an insulin-signaling pathway was analyzed by qRT-PCR in differentiated C2C12 myotubes after transfection with siCont or siCD36. (**C**) Western blot analysis and quantification of insulin- induced CD36, P-AKT (S473), total AKT (T-AKT), P-ERK (T202/Y204), and total ERK (T-ERK) expression levels in differentiated C2C12 cells after transfection with siCont or siCD36. Values are presented as the mean ± SEM of three independent experiments. * *p* < 0.05, ** *p* < 0.01, *** *p* < 0.001 versus the siCont group.

To investigate the role of CD36 in regulating the metabolic homeostasis of skeletal muscle cells, RNA-seq was performed in C2C12 myotubes with and without CD36 knockdown. Interestingly, the insulin signaling pathway was enriched by KEGG enrichment analysis (Figure 2A). Irs1, Irs2, and Pi3kr1 are key players in insulin signaling [20]. The expression levels of Slc2a4, Irs1, Irs2, and Pi3kr1 were downregulated in CD36-deficient C2C12 myotubes by qRT-PCR analysis (Figure 2B). Phosphorylation of AKT is thought to be a key step in insulin signaling in skeletal muscle [4,5]. The insulin-induced activation of AKT and ERK in CD36-deficient C2C12 myotubes was significantly lower than that of C2C12 myotubes (Figure 2C). Overall, CD36 deletion impairs insulin signaling in skeletal muscle cells in the absence of PA stimulation, suggesting its fundamental role in regulating insulin action.

### 3.3. Loss of CD36 Induces Mitochondrial Dysfunction in C2C12 Myotubes in the Absence of PA Loading

**Figure 3:**
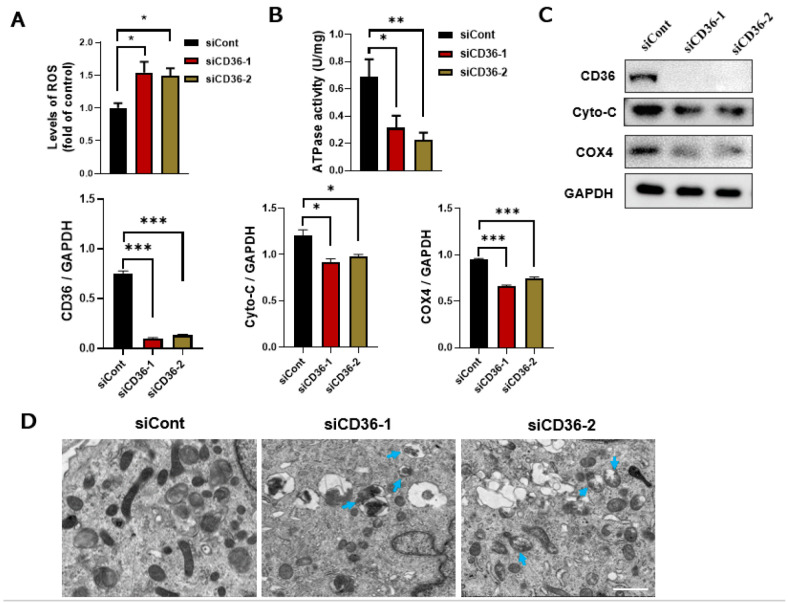
Loss of CD36 induces mitochondrial dysfunction in C2C12 myotubes without PA loading. (**A**) ROS content. (**B**) ATPase activity. (**C**) Western blot analysis and quantification of Cyto-C and COX4 protein expression in differentiated C2C12 cells after transfection with siCont or siCD36. (**D**) Ultrastructure changes in the mitochondria shown via electron microscopy in differentiated C2C12 cells after transfection with siCont or siCD36. Blue arrows indicate the injured mitochondria, (scale bar, 10 μm). Values are presented as the mean ± SEM of three independent experiments. * *p* < 0.05, ** *p* < 0.01, *** *p* < 0.001 versus the siCont group.

The underlying mechanism of CD36 on insulin signaling needs to be further explored. The overproduction of ROS can cause damage to the mitochondria and other cellular components, resulting in autophagy or apoptosis under high stress levels [21]. ROS content was measured to identify the function of CD36 deletion in ROS production. CD36 deletion significantly increased the ROS levels (Figure 3A), which might have impaired mitochondrial function. Mitochondrial function is expressed as changes in the protein level or the enzymatic activity of key mitochondrial components that promote oxidation, the mRNA levels of mitochondrial markers, and mitochondrial size and shape [22]. Reduced mitochondrial protein expression, a decreased oxygen consumption rate, and impaired ATP synthesis have been observed in people with obesity or IR [15,16]. ATPase is essential for mitochondrial function. Therefore, we investigated the possible role of CD36 deletion on ATPase activity. Results showed that ATPase activity was decreased in CD36-deficient C2C12 myotubes (Figure 3B). The expression levels of Cyto C and COX4 in CD36-deficient C2C12 myotubes were also significantly reduced compared to the controls (Figure 3C). A TEM was used to examine the effect of CD36 deficiency on mitochondrial ultrastructure. Significant damage was detected in the CD36-siRNA group, along with mitochondrial vacuolization, swelling, and mitochondrial crista rupture (shown by blue arrows in Figure 3D).

### 3.4. Loss of CD36 Enhances ER Stress in C2C12 Myotubes in the Absence of PA Loading

**Figure 4:**
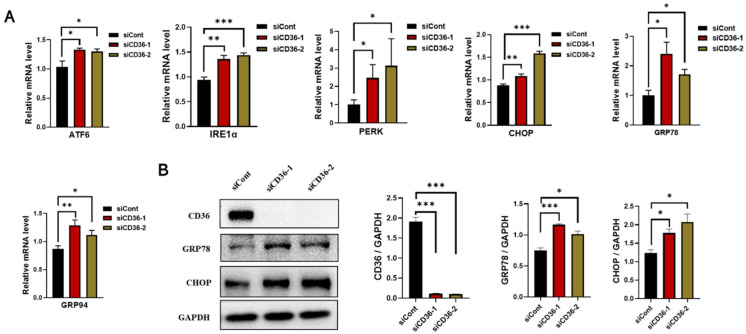
Loss of CD36 enhances ER stress in C2C12 myotubes without PA loading. (**A**) Expression of genes involved in ER stress (ATF6, IRE1α, PERK, CHOP, GRP78, and GRP94) as examined by RT-PCR in C2C12 myotubes with or without CD36 knockdown. (**B**) Western blot analysis and quantification of CD36, GRP78, and CHOP expression detected by western blot analysis in C2C12 myotubes with or without CD36 knockdown. Each protein expression level was normalized to that of the GAPDH gene. Values are presented as the mean ± SEM of three independent experiments. * *p* < 0.05, ** *p* < 0.01, *** *p* < 0.001 versus the siCont group.

IR in skeletal muscle is linked to increased ER stress markers [23]. The expression of ER stress markers was examined to explore the role of CD36 deletion in ER stress. The results of real-time PCR revealed a significant increase in the mRNA levels of ATF6, IRE1α, PERK, CHOP, GRP78, and GRP94 after CD36 deletion in C2C12 myotubes compared to the control group (Figure 4A). The significant effect of CD36 deletion on the expression of proteins related to the ER stress pathway was confirmed by examining the expression of GRP78 and CHOP by western blot analysis. The protein expression of GRP78 and CHOP significantly increased in CD36-deficient C2C12 myotubes (Figure 4B). Therefore, CD36 expression may play a key role in ER stress, which is partly responsible for impaired insulin signaling.

### 3.5. Loss of CD36 Protects against Insulin Resistance in the Presence of PA Overload

**Figure 5:**
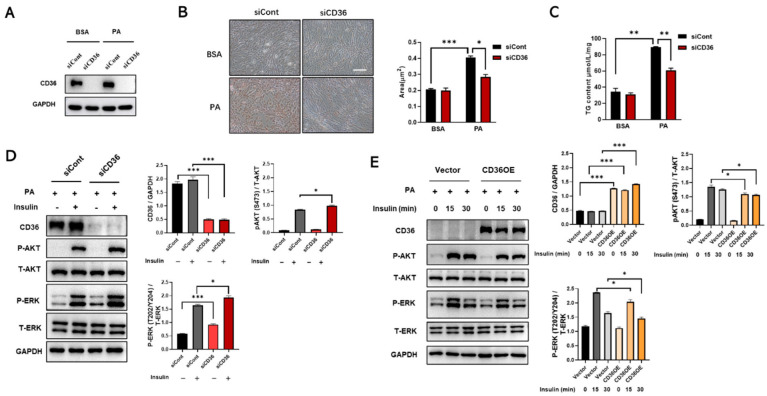
Loss of CD36 protects against insulin resistance in response to PA overload. Differentiated C2C12 myotubes transfected with siCont or siCD36 were treated with BSA or PA (200 μM) for 24 h. (**A**) The expression levels of CD36 were measured to assess the efficiency of knockdown. Lipid accumulations in differentiated C2C12 cells after CD36 siRNA or siCont transfection were examined by Oil Red O staining (**B**) and TG contents (**C**) under the conditions of BSA and PA stimulation. The area of lipid droplets (μm^2^) was calculated using the Image J system (scale bar, 10 μm). (**D**) Differentiated C2C12 myotubes transfected with CD36 siRNA or siCont were treated with PA (300 μM) for 15 min, and then treated with insulin (10 nM) for 30 min. Whole-cell lysates were subjected to WB analysis with antibodies against CD36, P-AKT (S473), total AKT (T-AKT), P-ERK (T202/Y204), total ERK (T-ERK), and GAPDH. (**E**) Vectoror CD36 overexpressing (CD36 OE) C2C12 myotubes were treated with PA (300 μM) for 15 min, and then treated with insulin (10 nM) for the indicated times. Whole-cell lysates were subjected to WB analysis with antibodies against CD36, P-AKT (S473), total AKT (T-AKT), P-ERK (T202/Y204), total ERK (T-ERK), and GAPDH. Values are presented as the mean ± SEM of three independent experiments. * *p* < 0.05, ** *p* < 0.01, *** *p* < 0.001 versus the control groups.

It is hypothesized that, in contrast to the absence of PA loading, loss of CD36 protects against insulin resistance in the presence of PA overload due to a reduction in lipid accumulation [24]. Therefore, the effect of CD36 deficiency on lipid accumulation under PA conditions was investigated. siRNA against CD36 was used for transfection to inhibit the CD36 mRNA and protein expression levels, which decreased remarkably (Figure 5A). After silencing CD36, lipid accumulation was alleviated in CD36-deficient C2C12 myotubes under PA stimulation (Figure 5B,C). Excessive intracellular lipid deposition is closely associated with the development of IR in skeletal muscle [15], so we further investigated the effect of CD36 on insulin signaling in the presence of PA overload. Our study showed that insulin-stimulated phosphorylation of AKT and ERK was increased in CD36-deficient myotubes compared to controls in response to PA overload (Figure 5D). In contrast, insulin-stimulated phosphorylation of AKT and ERK was decreased in CD36-overexpressing myotubes with PA overload (Figure 5E). These results suggested that CD36 negatively affects insulin signaling in the presence of PA overload, possibly related to the effects of intracellular lipid accumulation.

## 4. Discussion

The over-deposition of intramuscular triacylglycerol (IMTG) is closely related to muscle IR [25]. Considering the strong association between muscle IR and IMTG, we focused on CD36, which contributed to regulating LCFA uptake and oxidation in a coordinated manner [9]. Therefore, the expression levels of CD36 in the skeletal muscle tissues of obese humans and mice were first identified. Our results showed that the CD36 expression levels were upregulated in the skeletal muscle tissues of obese humans and mice compared to non-obese subjects. PA at concentrations of 0.4–1.0 mM can induce IR models in cultured skeletal muscle cells [26]. Thus, we established an in vitro model of C2C12 myotubes cultured in a PA medium as a way to further confirm the results in vivo. The results showed that CD36 protein was highly expressed in PA-stimulated myotubes. Collectively, these results suggested that CD36 might be associated with metabolic dysregulation in myotubes under the condition of PA overload.

Interestingly, the role of CD36 in regulating insulin signaling is complex [9]. It has been reported that this regulation may depend on the cellular response to different nutritional stresses [10]. Therefore, we explored the differential effects of CD36 deficiency on insulin signaling in C2C12 myotubes in the absence and presence of PA load. In the absence of PA stimulation under basal conditions, insulin-induced AKT phosphorylation expression levels were reduced in CD36-deficient C2C12 myotubes, which was also confirmed by RNA-seq and RT-PCR analysis, suggesting that CD36 deficiency disturbs insulin action and impairs insulin signaling, possibly because the presence of CD36 is essential for maintaining intracellular metabolic homeostasis (Figure 2). Emerging evidence supports our results that skeletal muscle insulin signaling is impaired in CD36-deficient mice [10]. Nevertheless, in our study, the contradictory result was observed in the case of PA overload. The results showed that CD36 deletion protected myotubes from insulin resistance in the presence of PA overload (Figure 5), probably because CD36 deletion reduced the uptake of excess LCFAs and alleviated lipid overaccumulation, thus improving the insulin signaling pathway [14]. This result was also confirmed by other in vivo studies that CD36 deletion protects mice from HFD-induced IR, obesity, and hypoglycemia [27]. The differences in CD36 promotion or prevention of IR may be related to FA-loading status and tissue specificity [28,29]. In addition, based on genetic studies, the effects of partial CD36 defects [30] are different from those of complete CD36 defects [13]. Our results showed that complete CD36 deficiency in the absence of PA load, much like CD36 overexpression in the presence of PA overload, may lead to metabolic complications. Thus, there may be a “metabolic protection” range or threshold effect for CD36 expression. Further studies are necessary to determine the molecular regulation of CD36 expression under different FA loading conditions and its contribution to tissue-specific functions to understand how CD36 affects specific obesity-related phenotypes and complications.

ROS-induced mitochondrial dysfunction and ER stress cause IR [31]. It is critical for cellular function to maintain proper mitochondrial function [18]. Recently, research interest in skeletal muscle mitochondrial function has increased because of the discovery of mitochondrial dysfunction in type 2 diabetes, including decreased muscular ATP synthesis [32,33], reduction in the activity of mitochondrial enzymes and expression of the electron transport chain [34,35], and aberrations in mitochondrial morphology and density [36,37]. Despite the controversy regarding IR, CD36 emerges as a biomarker for patients with type 2 diabetes and related complications. The role of CD36 in the regulation of mitochondrial function in the treatment of IR and type 2 diabetes needs to be determined [38]. However, the contributions of CD36 deficiency to the modulation of mitochondrial function have not been established. Our results demonstrated that CD36 deficiency induces a reduction in ATPase and several markers of mitochondrial genes, such as COX4 and Cyto C, and an aberration in mitochondrial morphology, suggesting impairment of mitochondrial function. A strong structural and functional connection has been observed between the mitochondrial network and ER [39,40]. It is reported that ER stress upregulated in CD36-deficient mice [10], which is consistent with our results that CD36 negatively regulated ER stress. It is hypothesized that in the absence of PA overload, CD36 is necessary for maintaining muscle lipid metabolism and its deficiency disturbs intracellular metabolic homeostasis (37,38), leading to increased ER stress in myotubes. The role of CD36 in regulating skeletal muscle ER stress in vivo needs to be further investigated.

## 5. Conclusions

**Figure 6:**
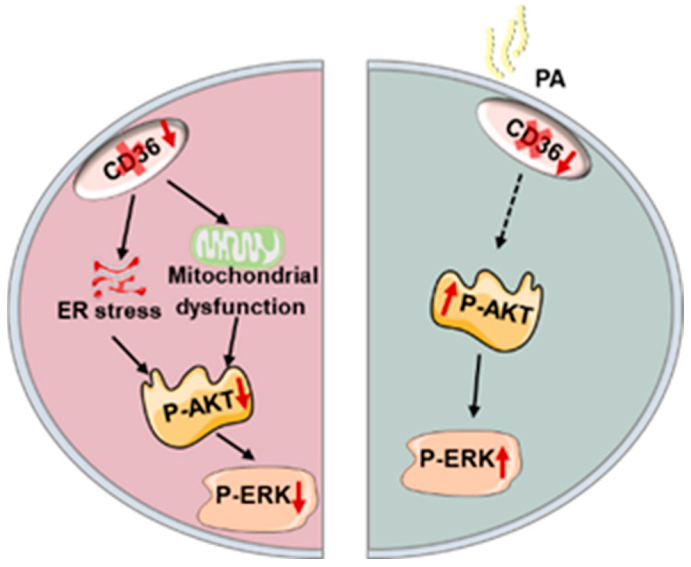
Schematic representation of the hypothesis that the differential role of CD36 in the regulation of muscle insulin response depends on palmitic acid load.

In summary, the effect of CD36 deficiency on insulin signaling is varied in C2C12 myotubes with or without PA overload. In the absence of PA load, CD36 deletion impairs insulin signaling, possibly by increasing mitochondrial dysfunction and ER stress. This suggests that CD36 is essential for the maintenance of physiological insulin action; however, CD36 deletion improves insulin signaling in the presence of PA overload due to a reduction in lipid accumulation (Figure 6). Although this work yielded a potential relationship between CD36 expression and insulin signaling in C2C12 myotubes, several limitations remain. First, additional key members of the insulin signaling pathway should be tested in the future and supplemented with physiological indicators to reflect the effects of CD36 more fully on insulin signaling activity. In addition, the dynamic impact of CD36 in the progression from IR to diabetes has not been elucidated. Finally, whether CD36 deficiency impairs insulin signaling by increasing mitochondrial dysfunction and ER stress lacks causal validation and requires further experimental proof. Uncovering these vital issues would be instrumental for designing novel CD36-targeted therapies, which would suppress or diminish IR and its harmful effects.

## Figures and Tables

**Table 1:** Sequences of primers for RT-PCR.

Gene	Sense (5′–3′)	Anti-Sense (5′–3′)
Slc2a4	GTGACTGGAACACTGGTCCTA	CCAGCCACGTTGCATTGTAG
Irs1	AGC GCG CCT GGA GTA TTA TGA GAA	GTC AGC CCG CTT GTT GAT GTT GAA
Irs2	AAA GTG GCC TAC AAC CCT TAC CCA	TCA TCG CTC TTG CAG CTA TTG GG
Pi3kr1	AAG GAG CTG GTG CTA CAT TAT C	CGC CTC TGT TGT GCA TAT ACT
ATF6	GACTCACCCATCCGAGTTGTG	CTCCCAGTCTTCATCTGGTCC
IRE1α	ACACTGCCTGAGACCTTGTTG	GGAGCCCGTCCTCTTGCTA
PERK	GCGTCGGAGACAGTGTTTG	CGTCCATCTAAAGTGCTGATGAT
CHOP	CTGGAAGCCTGGTATGAGGAT	CAGGGTCAAGAGTAGTGAAGGT
GRP78	ACTTGGGGACCACCTATTCCT	ATCGCCAATCAGACGCTCC
GRP94	TCGTCAGAGCTGATGATGAAGT	GCGTTTAACCCATCCAACTGAAT

## Data Availability

The data used to support the findings of this study are available from the corresponding author upon request.
